# Natural History of Influenza B Virus—Current Knowledge on Treatment, Resistance and Therapeutic Options

**DOI:** 10.3390/cimb46010014

**Published:** 2023-12-26

**Authors:** Ghayyas Ud Din, Kinza Hasham, Muhammad Nabeel Amjad, Yihong Hu

**Affiliations:** 1 CAS Key Laboratory of Molecular Virology & Immunology, Institutional Center for Shared Technologies and Facilities, Pathogen Discovery and Big Data Platform, Shanghai Institute of Immunity and Infection, Chinese Academy of Sciences, No. 320 Yueyang Road, Shanghai 200031, China; ghayyas@ips.ac.cn (G.U.D.);; 2 University of Chinese Academy of Sciences, Beijing 100040, China; 3 Sundas Molecular Analysis Center, Sundas Foundation Gujranwala Punjab Pakistan, Gujranwala 50250, Pakistan

**Keywords:** influenza B virus, treatment, therapeutic options, economic losses

## Abstract

Influenza B virus (IBV) significantly impacts the health and the economy of the global population. WHO global health estimates project 1 billion flu cases annually, with 3 to 5 million resulting in severe disease and 0.3 to 0.5 million influenza-related deaths worldwide. Influenza B virus epidemics result in significant economic losses due to healthcare expenses, reduced workforce productivity, and strain on healthcare systems. Influenza B virus epidemics, such as the 1987–1988 Yamagata lineage outbreak and the 2001–2002 Victoria lineage outbreak, had a significant global impact. IBV’s fast mutation and replication rates facilitate rapid adaptation to the environment, enabling the evasion of existing immunity and the development of resistance to virus-targeting treatments. This leads to annual outbreaks and necessitates the development of new vaccination formulations. This review aims to elucidate IBV’s evolutionary genomic organization and life cycle and provide an overview of anti-IBV drugs, resistance, treatment options, and prospects for IBV biology, emphasizing challenges in preventing and treating IBV infection.

## 1. Introduction

The influenza virus has a significant global impact, causing billions of cases, millions of severe cases, and hundreds of thousands of deaths annually [1]. Influenza is an acute respiratory infectious disease that is caused by the influenza virus. About 5% to 10% of adults and 20% to 30% of children are infected with the influenza virus every year [2]. Influenza viruses can be divided into three types, A, B, and C, according to the antigenicity of nucleoproteins, among which A and B are the primary pathogens that cause human influenza [3]. The frequency of localized epidemics caused by influenza B viruses has increased worldwide in recent years [4,5]. Influenza viruses rapidly evolve with antigenic diversity, resulting in new hemagglutinin and neuraminidase proteins. Antigenic shifts can cause pandemics, and antigenic mutation contributes to global impact [4]. Vaccinations since the 1960s have reduced the number of influenza cases, and antiviral drugs (neuraminidase and viral polymerase inhibitors) treat influenza B. The vaccine’s efficacy is approximately 60% in a good season. In case of mismatch with current circulating strains, its effectiveness decreases from 10% to 20% [6]. However, the vaccine’s estimated overall efficacy stands at 38% (62% against the flu A[H1N1] pdm09 viruses, 22% against flu A[H3N2] viruses, and 50% against the influenza b virus) [7]. Several factors influence the efficiency of influenza vaccines [7]. One of the major factors is that the genes in various strains of influenza viruses undergo constant mutations, altering the surface proteins and enabling the virus to evade the host’s immunological response. This phenomenon, termed antigenic variation, allows the virus to avoid detection, thereby increasing its virulence by circumventing the body’s pre-existing immune defense. In cases where the mutation is gradual and minor, the host (human) may manage to trigger an immunological response by producing antibodies. This gradual transformation over time is called antigenic drift [8]. Other factors influencing vaccine efficacy include the recipient’s age and health, the types and subtypes of circulating virus strains, and the degree of homology between the circulating virus and those included in the vaccine [7]. However, antiviral drug resistance is a serious concern. This review discusses the evolution, genomic organization, life cycle, anti-IBV drugs, resistance, and future therapies of the influenza B virus.

## 2. Epidemiological Histories

**Figure 1:**
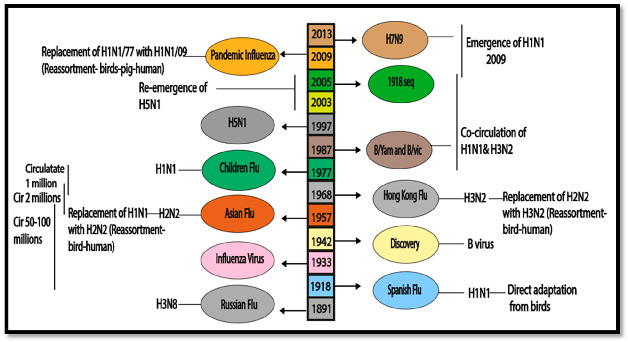
Epidemiological history and co-circulation of influenza virus.

In this section, we summarize the historical background of influenza virus circulation and its impact on epidemiology, immunology, and vaccination. Since 1889, several Influenza A virus pandemics have significantly affected the human immune system, with the H3N8 and H1N1 viruses presumed responsible for the 1889 and 1918 pandemics (Figure 1) [9].

The H1N1 virus, causing the deadliest pandemic in modern history with 50 to 100 million deaths worldwide, was identified through sequencing in 1997 and revived in 2005 [10,11]. Following the 1918 pandemic, H1N1 viruses continued circulating in humans, and the 1957 Asian pandemic introduced the H2N2 virus, causing approximately 2 million deaths [12]. The emergence of the H3N2 virus during the Hong Kong pandemic in 1968 led to the replacement of the H2N2 subtype due to population resistance to sub-immuno-dominant components [13]. In 1977, the H1N1 virus re-emerged, co-circulating with H3N2 until the sixth pandemic in 2009, caused by the introduction of a unique H1N1 lineage [14]. Zoonotic strains, such as H5N1, H6N2, H7N1, H7N7, and H9N2, have been reported to infect humans [15]. In 2013, H7N9 posed a serious threat to human health in China [16]. Influenza B viruses have been co-circulating since 1891, leading to a complex immunological environment. The WHO identified B/Yamagata/16/1988 (B/Yamagata lineage) and B/Victoria/2/1987 (B/Victoria lineage) by monitoring in the late 1980s [17].

The influenza virus pandemic also leads to economic loss. The present study concluded a short comparative analysis between China and the USA (Table S1 and Figure S1). Both China and the USA incur substantial economic losses due to influenza. China’s larger population and higher population density can lead to rapid transmission and significant healthcare costs and absenteeism [18]. The lower population density does not prevent substantial losses in the USA, with higher per capita healthcare costs and productivity impacts. Factors such as vaccination rates, healthcare infrastructure, and public health policies influence the extent of these economic burdens in each country [19].

## 3. Organization of Genome

**Figure 2:**
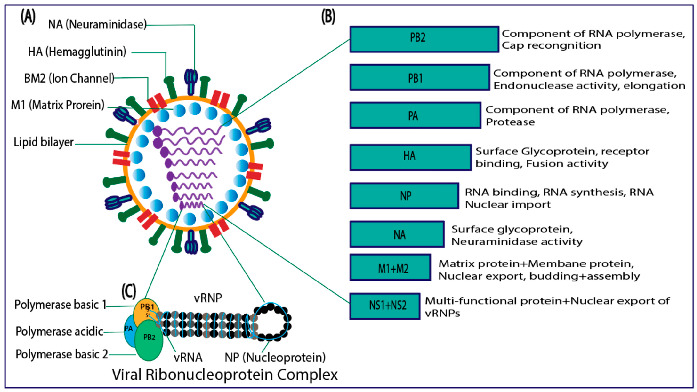
(**A**) Structure of influenza B virus. (**B**) Genome organization. (**C**) viral ribonucleoprotein complex.

IBVs are from the Orthomyxoviruses family, which comprises negative-sense single-stranded RNA with a segmented genome. IBVs include eight segments and are encodes for glycoproteins, matrix proteins, BM2 ion channels, polymerase proteins, nuclear export proteins, nonstructural proteins, and nucleoproteins (Figure 2) [20,21]. None of the IAV “accessory” proteins, such as PB1-F2 or PA-X, are encoded by IBVs. In general, there are significant differences between IAV and IBV proteins in terms of the length, function, and composition of amino acids [22]. IBV proteins are discussed below.

### 3.1. The Hemagglutinin

IBV’s HA protein facilitates virus attachment to host cells through interactions with sialic acids [23]. It can bind to both α-2,3- and α-2,6-linked sialic acids. Structurally, IBV HA forms a functional homo-trimer with a globular membrane-distal region and an extended membrane-proximal region. Notably, there are significant differences in the receptor-binding site (RBS) between IBV HA and IAV HA, particularly at position 95, where IBV has phenylalanine, while IAV has tyrosine [17]. Phenylalanine at position 95 in IBV HA contributes to its lower affinity and ability to bind human-like and avian-like receptor analogues. Mutations that replace phenylalanine with tyrosine improve receptor binding similar to IAV and reduce adherence to avian-like receptor analogues [21]. Unlike IAV, the HA protein of IBV does not play a significant role in pathogenicity [20].

### 3.2. The Neuraminidase

IBV’s NA protein, like IAV NA, cleaves sialic acid to release new virions from the cell surface. Despite a 30% sequence homology, IBV NA has structural similarities to IAV NA. IBV NA forms a tetramer with a Ca2+-binding region and two glycosylation regions (Asn143 and Asn283) in each monomer. Asn143 is conserved across influenza viruses, while Asn283 is specific to IBV [22]. The active site of NA consists of 19 conserved residues, including R118, D151, R152, R224, E276, R292, R371, and Y406, which interact with sialic acids, and E119, R156, W178, S179, D198, I222, E227, H274, E277, N294, and E425, which maintain its structural integrity. Neuraminidase inhibitors (NAIs) approved for treating influenza may not effectively target the IBV [23].

### 3.3. The NB Protein

IBV’s segment 6 encodes NB, overlapping with the NA gene. NA’s start codon follows NB’s start codon by four nucleotides [20]. NB is a glycoprotein of around 100 amino acids in the virion, with modified N-glycosylation regions containing N-acetyl-glucosamine residues [20]. The palmitoylation of C-terminal residues is required for NB’s cell surface transport [24]. Previous studies suggest NB’s potential ion channel functionality based on comparisons with influenza A virus’s M2 protein [25]. Assessing NB’s electrophysiological properties using lipid bilayers is challenging due to hydrophobic proteins and peptides exhibiting channel-like behavior [26]. Studies questioning NB’s channel activity contradict its assumed role as an ion channel protein, despite amantadine’s inability to inhibit influenza B virus growth [20]. NB’s purpose and function have remained enigmatic for over 30 years, and understanding it may contribute to the development of innovative live attenuated vaccines [27].

### 3.4. The Matrix Protein (BM1)

IBV’s M1 protein, encoded in segment 7, is vital in the B/Ann Arbor/1/66 master donor strain of the live attenuated influenza vaccine (LAIV) and IBV transformation in mice [28]. The precise role of M1 in the IBV life cycle is unclear, but it shuttles between the nucleus and cytoplasm, possessing nuclear export signals and a nuclear localization signal (NLS) [29]. Further research is required to elucidate the specific functions of BM1 and its resemblance to IAV M1.

### 3.5. The BM2 Ion Channel

BM2, encoded in segment 7, enters virions via the trans-Golgi network (TGN). It integrates vRNP similar to AM2, supporting virus viability. BM2 exhibits ion-conducting activity and shares similarities with AM2 in endosome acidification and Golgi pH adjustment. Its crystal structure reveals coiled-coil tetramers with polar-pore-lining residues, rendering adamantanes ineffective against BM2. Recent studies suggest a potential role of BM2 in inhibiting p53-mediated apoptosis and transcription. BM2 is expressed from a single mRNA molecule that translates both BM2 and M1 coding sequences with overlapping termination and start codons. The 45 nucleotides preceding the pentanucleotide sequence (UAAUG) within the M1-coding region are critical for BM2 expression. The upstream sequence of the pentanucleotide shows homology with the small ribosomal subunit (18S component), and translation initiation factor eIF3 and the mRNA-rRNA association are essential for BM2 expression. Initially discovered in BM2, this termination–reinitiation process is observed in other viruses, including caliciviruses [30,31,32,33,34].

### 3.6. The Nucleocapsid Protein

IBV nucleoprotein (BNP) differs significantly from IAV nucleoprotein (ANP) in structure and function. BNP lacks ANP’s NLSs and has a longer N-terminus with a unique 50 amino acid region. BNP’s structure includes a head, body, and long tail, with essential lysine clusters for RNA binding in the charged loop (residues 125–149). BNP’s extensible tail loop facilitates homo-oligomer formation, similar to ANP [22,35]. A conserved salt bridge (R472-E395 in BNP and R416-E339 in ANP) stabilizes loops and promotes homo-oligomerization, making it a potential therapeutic target [36]. BNP’s N-terminus is involved in nuclear localization through the K44RTR47 motif, and its removal affects virus replication and transcription [37].

### 3.7. The NS1 Protein

IBV’s NS1 protein (segment 8) is vital for virus replication [38]. It has an N-terminus (residues 1–90), C-terminus (residues 120–281), and associated domain (residues 91–119) [39]. NS1B localizes in the cytoplasm and interacts with importin α3, SC35 nuclear factor, and RNA. Unlike IAVs, NS1B does not hinder mRNA export but affects antiviral and host mRNA export [40]. NS1B inhibits IFN-αβ activation and nuclear translocation, increasing IFN expression and promoter activation in IBV when absent [41]. NS1B acts as an IFN response inhibitor, complementing NS1A IAV growth [42]. The C-terminus inhibits IFN promoter activity, while the N-terminus limits PKR kinase activation. NS1B blocks interferon-stimulated ISG15 protein, contributing to IBV host restriction. NS1B and NS1A have distinct roles in evading the innate immune response, with ISG15 playing a critical antiviral role against IBV. NS1B-deleted viruses attenuate in IFN-insufficient Vero cells, indicating additional functions of NS1B [43].

### 3.8. The NEP or NS2 Protein

IBV’s NEP facilitates nuclear export with a specific signal sequence in its N-terminus. It accumulates in the nucleus during late infection and then relocates to the cytoplasm near the plasma membrane for virion budding. NEP is a component of the vRNP complex in virions, and in contrast to IAV, NEP directly interacts with vRNP and BM1 in IBV. While the exact role of IBV NEP remains uncertain, it likely shares a similar function to its IAV counterpart [22,44].

### 3.9. Noncoding Sequences

Influenza viruses have noncoding regions that contain packaging signals and act as viral promoters for replication. These regions are crucial for vRNA replication in IBV [45]. Highly conserved sequences near the termini, such as 5′-AGCAGAAGC-3′ and 3′-TCATCxTTGT-5′, form secondary RNA structures through complementarity. Noncoding sequences, varying between segments but consistent within each segment, are located alongside the coding sequences in the long terminal regions. Understanding these noncoding sequences is essential for reverse genetics systems and creating chimeric IAVs/IBVs for potential vaccines [5,10,46].

## 4. Life Cycle of Influenza Virus

**Figure 3:**
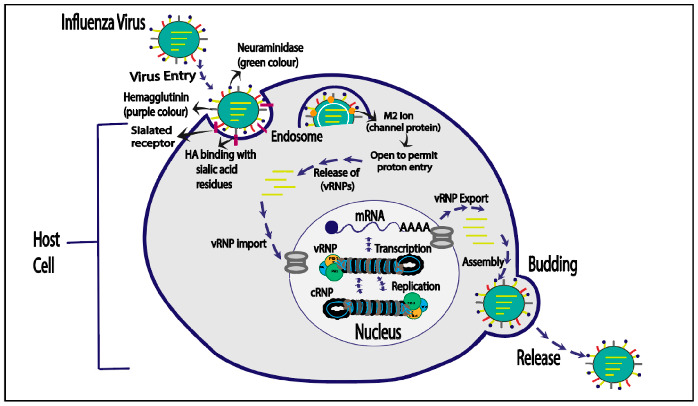
Life cycle of influenza virus.

The influenza virus life cycle begins with HA binding to sialyl oligosaccharides on the host cell surface, leading to viral attachment. The virus enters the host cell through receptor-mediated endocytosis and undergoes fusion with the endosomal membrane, triggered by a pH change and the M2 ion channel. This releases uncoated vRNP complexes into the cytosol. Once inside the nucleus, vRNP complexes replicate and transcribe vRNA, generating viral proteins. The newly produced vRNAs are transported to the cell membrane, cleaved by neuraminidase, and released as influenza, of which a virion is shown in Figure 3 [10].

### 4.1. Influenza Virus Entry

The influenza virus life cycle initiates with viral attachment to the host cell surface, facilitated by the interaction of HA with sialyloligosaccharides on the cell membrane. Using receptor-mediated endocytosis, the virus enters the host cell, where it is contained inside an endosome. The low pH in late endosomes triggers a structural change in HA, controlled by the M2 ion channel. The process culminates in a fusion of the viral and endosomal membranes. This results in the release of uncoated viral ribonucleoprotein (vRNP) complexes into the host cell cytoplasm. Neuraminidase cleaves freshly generated vRNPs after they have been transported to the cytoplasm and integrated by viral proteins at budding sites within the host cell membrane. These vRNPs are then discharged as influenza virions. (Figure 3) [47].

### 4.2. Viral Ribonucleoproteins’ (vRNPs) Entry into the Cell Nucleus

The viral RdRp is composed of three different types of viral proteins (PA, PB1, and PB2). The nuclear localization signals (NLSs) of these proteins have the potential to bind to the cellular nuclear import apparatus, which makes it easier for the proteins to enter the nucleus. The most important NLS for the vRNP nuclear entrance is still unknown. Notably, the nuclear import happens via a Crm1-dependent pathway, which involves binding several karyopherins like importin α and β [48].

### 4.3. Replication and Transcription

The influenza virus, with negative-sense RNA strands, undergoes transcription by converting the genome into positive-standard RNA, acting as a template for the synthesis of viral RNA. Genome replication occurs without primers as the virus’s RNA-dependent RNA polymerase (RdRp) begins internal synthesis. Partial inverse complementarity at the genome’s extreme ends facilitates the formation of corkscrew structures. The complete replication mechanism remains unknown despite the presence of di-nucleotide base pairs [49]. Mature cellular messenger RNAs (mRNAs) have a poly(A) tail and a 5′ methylated cap. Although vRNPs lack 5′ caps, they possess poly(A) tails. The absence of a 5′ cap in the viral genome puzzled researchers until they discovered that the viral mRNAs had a poly(A) tail and a 5′ methylation cap. Further research revealed that the 5′ methylation caps of viral mRNAs are associated with cellular mRNAs, leading to the “cap-snatching” method [50]. The viral RdRp, composed of PA, PB1 and PB2, utilizes PB2 to cleave the 3′ caps’ regions with 10–15 nucleotides. The viral RdRp initiates transcription using cellularly bound RNA fragments [51]. The cellular cap synthesis complex activates when transcription initiation involves cellular RNA polymerase II (Pol II) attaching to DNA, with the phosphorylation of serine five on Pol II’s C-terminal repeat domain (CTD). Research indicates that the influenza RdRp prefers binding to this form of Pol II, suggesting a potential site for “cap snatching” [52].

### 4.4. vRNPs Export

vRNPs most likely use the CRM1-dependent method to exit the nucleus through nuclear pores. In the lack of clear GTP hydrolysis activity, direct contacts between NP and CRM1 suggest an unusual export mechanism. M1’s C-terminal end directly binds to vRNPs, and its N-terminal region has a putative NLS connected to vRNP import. M1’s N-terminal segment can bind to NEP, hiding the NLS. NEP forms a compound with CRM1 after GTP hydrolysis, which may indicate a “daisy-chain” export route for vRNPs from the nucleus [52].

### 4.5. Budding Host Cell Plasma Membrane

After the exit of vRNPs from the nucleus, the virus assembles and leaves the host cell by utilizing the plasma membrane. Essential viral proteins (HA, NA, and M2) are crucial for particle generation. Elongated particles result from modifying the M2 tail. M1, beneath the lipid bilayer, aids in particle closure and budding. Sialic acid cleavage by NA is essential for viral particle release from the plasma membrane [47,53,54].

## 5. Treatment: Anti-IBV Drugs and Resistance

**Figure 4:**
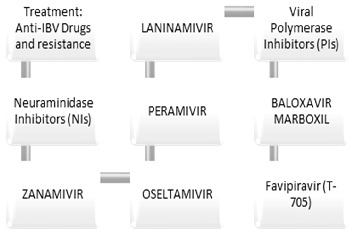
Treatment anti IBV drugs and resistance.

Neuraminidase inhibitors, viral polymerase inhibitors, and antiviral drugs are likely aimed at reducing the severity of symptoms and limiting the spread (Figure 4).

### 5.1. Neuraminidase Inhibitors (NIs)

**Figure 5:**
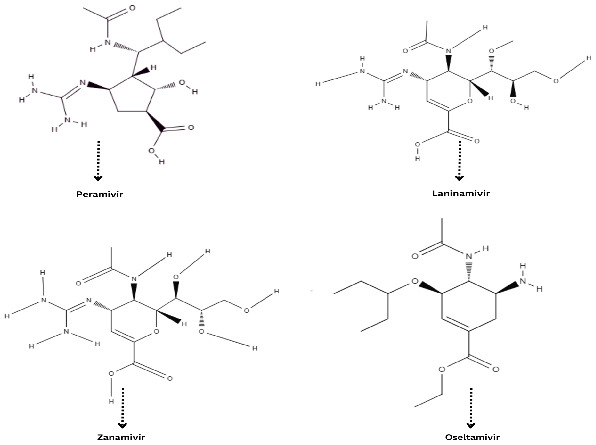
Chemical structure of influenza B virus neuraminidase inhibitor.

In 1948, the idea of using neuraminidase inhibitors (NI) as antiviral drugs emerged. After extensive research, zanamivir (Relenza^®^) and oseltamivir (Tamiflu^®^) were developed in 1999 and 2000, respectively. More recent additions to this class include peramivir (Rapivab^®^, Rapiacta^®^, PeramiFlu^®^, and Alpivab^®^) in 2010 and laninamivir (Laninamivir^®^) in 2014 [55]. Neuraminidase inhibitors and resistance are described below, the structural chemical composition is shown in Figure 5, and the resistance-conferring mutation of the Influenza B virus to neuraminidase and polymerase inhibitor is shown in Table 1 [55].

### 5.2. Zanamivir (Relenza^®^)

Zanamivir, a 4-deoxy-4-guanidino analog of DNA, is administered through oral inhalation due to its limited oral bioavailability (2–10%). It is primarily deposited in the mouth and throat, important sites of influenza infection [56]. Zanamivir is recommended for treating influenza in patients aged 7 years and above, with symptoms lasting no more than 2 days. A 1% solution is advised for children above 6 months old. In severe cases or when other treatments fail, the intravenous form of zanamivir (Dectova^®^) may be used. Zanamivir is hydrophilic and eliminated in its unmodified form by the kidneys [57]. In IBV, zanamivir’s effectiveness is reduced by specific NA mutations (R371K, G108E, D197N, I221T, T146K, and T146P). Combing T146P with a second mutation at N169S further decreases the efficacy of neuraminidase inhibitors. Additional NA mutations (H101L, A200T, D432G, H439P, and H439R) also reduce the effectiveness of neuraminidase inhibitors [58].

#### Contraindications/Adverse Events

Zanamivir is not recommended for pregnant or breastfeeding patients and those with hypersensitivity to any component of the preparation. Rare adverse effects of zanamivir include nasal symptoms, headache, dizziness, disorientation, bronchospasm, breathing impairment, skin rashes angioedema, and urticaria [59].

### 5.3. Oseltamivir

Oseltamivir is a prodrug that is converted into active oseltamivir carboxylate. It is recommended for treating influenza symptoms during outbreaks, with up to 30% reduction in severity if treatment is started within 2 days. Oseltamivir has higher bioavailability than zanamivir [60]. Influenza A/H3N2 and influenza B viruses with neuraminidase inhibitor resistance show reduced growth and transmission potential. Resistance usually develops after prolonged treatment but dissipates upon discontinuation. The R292K mutation confers resistance to both zanamivir and oseltamivir. Several influenza A and B phenotypes have reduced susceptibility to oseltamivir and zanamivir [61]. Mutations in the neuraminidase (NA) gene, such as R371K, H273Y, I221T, K152M, D197N, T146K, T146P, N169S, G108E, and A200T, are associated with reduced sensitivity to oseltamivir. Resistance levels can vary between the B-lineages B/Victoria and B/Yamagata [62].

#### Contraindications/Adverse Events

Patients with hypersensitivity to oseltamivir or any of its ingredients, impaired kidney function, fructose intolerance, chronic cardiac illness, severe hepatic impairment, and respiratory disease should not use oseltamivir. Nausea, vomiting, abdominal discomfort, diarrhea, dyspepsia, headache, lethargy, sleeplessness, disorientation, conjunctivitis, epistaxis, and skin rashes are all common side effects of oseltamivir. Stevens–Johnson syndrome, toxic epidermal necrolysis, hepatitis, and mental abnormalities in children recorded in extremely uncommon circumstances [59].

### 5.4. Peramivir

Peramivir effectively inhibits the neuraminidase (NA) of influenza A and B viruses, including highly virulent strains like H5N1 [63]. It is marketed under different names in different regions, such as Rapiacta^®^ in Japan (2010 approval), PeramiFlu^®^ in South Korea (2010 approval), Rapivab^®^ in the US (2014 approval), and Alpivab^®^ in the EU (2018 approval, subsequently removed from the market) [64]. Rapivab^®^ is the only FDA-approved intravenous neuraminidase inhibitor (NAI) for treating acute and mild influenza, offering the convenience of a single dose [65]. Certain NA mutations, including T106P, G104R/G, G145E, I221T, D197N, A245T, K360E, A395E, D432G, G145R, Y142H, H273Y, T146K, T146P, N169S, G247D, I361V, G108E, H101L, A200T, D432G, and H439P, reduce the efficacy of peramivir [65].

#### Contraindications/Adverse Events

People who have a history of severe allergic-like reactions to any of the components of peramivir or other neuraminidase inhibitors (NAIs) should not take peramivir. Notably, severe allergic hypersensitivity events have been reported in connection with various nucleoside analog inhibitors, including zanamivir and oseltamivir [66]. Adverse events including severe allergic responses and hypersensitivity to medications or chemicals have been linked to erythema multiform, anaphylaxis, Stevens–Johnson syndrome, pregnancy, breastfeeding, gastrointestinal disturbances, central nervous system effects, hematological effects, and allergic reactions [67].

### 5.5. Laninamivir

Laninamivir (Inavir^®^) is a neuraminidase inhibitor used in Japan. It is a prodrug that is converted to its active form in the respiratory tract. Orally administered, laninamivir exhibits prolonged high concentration in the lungs, exceeding the IC50 for influenza virus for up to 240 h. It binds effectively to viral neuraminidase with a single inhalation dose of 40 mg, which is sufficient for antiviral effects. Resistance to laninamivir has been associated with NA mutations, including T146P, N169S, T146K, and A200T in influenza B virus strains [68].

#### Contraindications/Adverse Events

Laninamivir is contraindicated in pregnant or breastfeeding individuals and those with hypersensitivity or allergy. Additionally, it is not recommended for use in patients with severe renal impairment [69]. Adverse events include gastrointestinal symptoms, respiratory symptoms, headache, dizziness, allergic reactions, and neuropsychiatric events [69].

### 5.6. Viral Polymerase Inhibitors (PIs)

PB1, PB2, and PA are the three subunits of the influenza virus’s polymerase, which is essential for generating viral RNA. Two viral polymerase inhibitors are currently in use, Baloxavir and Favipiravir. Baloxavir inhibits the PA component, whereas favipiravir blocks PB1. So, both drugs prevent the mechanism necessary for the virus’s life cycle [70].

#### 5.6.1. Contraindications/Adverse Events

Baloxavir Marboxil is not recommended for use in pediatric patients under the age of 12 years and those with hypersensitivity, pregnancy and breastfeeding, metabolic disturbances, and in rare cases, difficulty breathing. Adverse events include allergic reactions, changes in blood cell counts, hepatic and renal effects, and central nervous system effects [71].

#### 5.6.2. Baloxavir Marboxil

Baloxavir (Xofluza^®^) is a novel anti-influenza medication that is metabolized into baloxavir acid. It was approved in Japan in 2018 and later in other countries, including the European Union in 2021. Baloxavir is administered orally and has a long half-life, requiring a single dose for treatment. It is effective against various influenza A and B strains, including oseltamivir-resistant strains and subtypes A/H5N1 and A/H7N9. However, resistance can develop due to substitutions at position PA I38T/M/F. Before approval, a limited number of viruses with mutations at PA residue 38 were observed. These findings highlight the impact of I38T and I38M PA substitutions in reducing susceptibility to Baloxavir in existing influenza B strains [72,73].

#### 5.6.3. Favipiravir (T-705)

Favipiravir (Avigan^®^) is an antiviral drug that inhibits RNA-dependent RNA polymerases. It is administered orally and intravenously to treat influenza A and B in Japan, particularly during pandemics caused by resistant strains. A recent study identified the K229R mutation in the PB1 subunit of the influenza virus polymerase, reducing favipiravir susceptibility in vitro and in a cell culture. However, a compensating mutation (P653L) can restore viral fitness. The clinical implications of these mutations are still unknown [73].

**Table 1:** Resistance-conferring mutation of influenza B virus to neuraminidase and polymerase inhibitor.

		Resistance-Conferring Mutation of Influenza B Virus to Neuraminidase and Polymerase Inhibitor						
Target Site Inhibitor	Generic Name	Routes of Administration	DrugBank Accession Number	Period of Time	Mutations Conferring Resistance	Worlds Region	Techniques Used	Ref
Neuraminidase Inhibitors	Zanamivir	Respiratory (inhalation)	DB00558	2004–2005	R371K	Hong Kong	Chemiluminescent NAI assay, IC50 analysis, reverse transcription-PCR, and sequence analysis	[60]
				2014–2015	D197N, I221T	Australia, USA, China, Ukraine, Japan, Russia	None (Report from global update)	[61]
				2018–2020	T146K	Philippines	NA activity assay, fluorometric NAI assay	[62]
					T146P, N169S, G247D, I361V, G108E, H101L, A200T, H439P	Malaysia	NA activity assay, fluorometric NAI assay	[62]
	Oseltamivir	Oral	DB00198	2004–2005	R371K	Hong Kong	Chemiluminescent NAI assay, IC50 analysis, reverse transcription-PCR, and sequence analysis	[60]
				2011	H273Y	Canada	Chemiluminescent NAI assay, IC50 analysis, sequence analysis	[63]
				2014–2015	I221T(NA), K152M(NA), D197N(NA)	Honduras, Bangladesh, Australia USA, China Ukraine, Japan, Russia	None (Report from global update)	[61]
				2016–2019	T146K, T146P, T146P, N169S, G108E, A200T	Asia	NA activity assay, fluorometric NAI assay	[62]
	Peramivir	Intravenous	DB06614	2014–2015	T106P, G104R/G, G145E, I221T, D197N, I221T	Japan, Honduras, Australia, USA, China Ukraine, Russia	None (Report from global update)	[61]
				2009–2012	I221T, A245T, K360E, A395E, D432G, G145R + Y142H	Asia, Africa, Oceania	Chemiluminescent NAI assay, IC50 analysis, reverse transcription-PCR, and sequence analysis	[65]
				2011	H273Y	Canada	Chemiluminescent NAI assay, IC50 analysis, reverse transcription-PCR, and sequence analysis	[62]
				2016–2020	T146K, T146P, N169S, G247D, I361V, G108E, H101L, A200T, D432G, H439P	Asia	NA activity assay, fluorometric NAI assay	[62]
	Laninamivir	Respiratory (inhalation)	DB12791	2019	T146P, N169S, T146K	Malaysia	NA activity assay, fluorometric NAI assay	[62]
				2018	T146K	Philippines	NA activity assay, fluorometric NAI assay	[62]
				2019	A200T	Malaysia	NA activity assay, fluorometric NAI assay	[62]
Polymerase Inhibitor	Baloxavir Marboxil	Oral	DB13997	2020	PA-I38T, PA-I38M	Reported only in vitro	Minigenome assay for polymerase activity, replication kinetics experiments, In vitro genetic stability	[73]

## 6. Targeted Therapies

**Figure 6:**
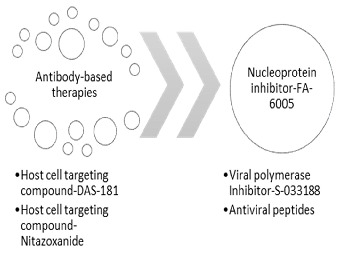
Targeted therapies.

Precise therapies aiming to disrupt specific disease-related molecules are shown in Figure 6.

### 6.1. Antibody-Based Therapies

Passive immune defense using broadly neutralizing antibodies (bnAbs) is a promising strategy for treating viral infections [74]. Researchers have generated bnAbs against the influenza B virus’s HA protein, aiming to provide protection against multiple strains. Antibodies like CR8033 and C12G6 target the receptor binding site (RBS) on HA, blocking viral entry, but they may not effectively prevent entry of all influenza B strains [75]. Developing potent bnAbs to prevent viral entry is crucial for combating emerging influenza B strains. Another study produced IgM and IgG bnAbs targeting the RBS of the influenza B virus, with C7G6-IgM showing strong hemagglutination inhibition (HI) and neutralization against all tested strains. However, further validation and characterization of these antibodies are needed to fully understand their antiviral actions and enable the development of universal vaccines against influenza B [76].

### 6.2. Host Cell Targeting Compound—DAS-181

Channel blockers, neuraminidase inhibitors, and polymerase inhibitors are approved treatments for influenza. They are known as direct-acting drugs (DADs). DAS181 (FludaseTM) is an inhalable dry powder with broad-spectrum antiviral properties against respiratory viruses, including influenza and parainfluenza. It contains a chimeric protein that breaks sialic acid bonds on host cells, preventing viral attachment. DAS181 has shown efficacy against parainfluenza in immunocompromised patients. Phase 2 clinical trials in the USA are currently evaluating its impact on influenza viral load, safety, and tolerability [10].

### 6.3. Host-Cell-Targeting Compound—Nitazoxanide

Nitazoxanide (NTZ) is a broad-spectrum antiviral drug that rapidly converts to tizoxanide (TIZ) upon oral administration [75]. Initially developed for antiprotozoal/helminth purposes, NTZ is now approved for treating Cryptosporidium infections [77]. It also exhibits effectiveness against bacterial and viral infections, including respiratory viruses (e.g., influenza), hepatitis B and C viruses, and gastrointestinal viruses (e.g., rotavirus) [78].

NTZ/TIZ acts through various mechanisms against these viruses. It disrupts viral HA assembly in influenza, inhibits VP7 production in rotavirus, and activates innate immunity via PKR activation in hepatitis C [79]. In vitro studies confirm the antiviral activity of NTZ/TIZ against multiple influenza A (H1, H3, H5, and H7) and B viruses. Combining NTZ/TIZ with oseltamivir, a virus-targeting antiviral, demonstrates synergistic effects against oseltamivir-resistant avian and human influenza A virus. However, the efficacy of NTZ treatment for influenza in animal models remains unknown [80].

### 6.4. Nucleoprotein Inhibitor—FA-6005

FA-600 is a potent nucleoprotein inhibitor that shows broad antiviral activity against human influenza A and B viruses. In vitro studies have demonstrated its ability to inhibit viral replication and impede vRNP movement within cells at multiple stages of the viral life cycle. FA-600 disrupts crucial steps, including attachment, penetration, replication, transcription, and exportation. The molecular target of FA-600 is amino acid residue 41, where it binds to the vRNP complex. This interaction reduces the export of NP or vRNP, hampers the movement of circulating ribonucleoproteins (RNPs) in the cytoplasm, interferes with viral uncoating and vRNP import, and accelerates the disruption of virion budding (Figure 3) [81].

### 6.5. Viral Polymerase Inhibitor—S-033188

S-033188 is a prodrug that undergoes metabolism to its active form, S-033447. This small molecule inhibits the cap-dependent endonuclease of influenza A and B viruses. In a mouse model, a single administration of S-033188 reduced virus burden by approximately 2 logs and improved survival compared to oseltamivir-treated mice. In vitro, S-033447 demonstrated high efficacy against a range of influenza A and B viruses. In a phase II trial involving 400 adult patients with uncomplicated influenza, three doses of S-033188 (10 mg, 20 mg, and 40 mg) significantly reduced symptom duration and viral titers at 24 and 48 hours compared to placebo. A phase III clinical study (NCT02949011) is currently underway to evaluate the efficacy of a specific dose of S-033188 compared to a placebo or oseltamivir in individuals at high risk of influenza complications [82].

### 6.6. Antiviral Peptides

Peptides are biologically active compounds that have evolved as an early chemical defense against various pathogens [83]. They are being considered as potential drugs due to their selectivity, specificity, and lower side effects compared to traditional drugs. However, their use is limited by rapid degradation and removal from the bloodstream. Pulmonary delivery is a promising approach to deliver therapeutic peptides to target cells, bypassing digestive enzymes [84]. The modification of peptide structures can enhance their therapeutic targeting [85]. Antiviral peptides prevent viral attachment and fusion, disrupt the viral coat, and inhibit viral polymerase. Intracellular delivery remains challenging for peptide-based inhibitors that target viral polymerase or assembly. The repeat administration of the same peptide may trigger unwanted immune responses. While there are potential issues to address, careful planning and further research can unlock the potential of antiviral peptides as a novel class of broad-spectrum medications [83].

## 7. Concluding Remarks

A common characteristic of viruses and every other organism is genetic drift. It serves as the basis for adapting to a changing environment and natural selection. RNA viruses have a faster rate of mutation as compared to DNA. It occurs because RNA polymerase lacks certain mechanisms to verify and rectify nucleotide attachment during replication. Influenza viruses are a significant public health concern due to their ability to undergo genetic changes through processes like mutations, reassortment, and recombination. These changes can lead to adaptations to new hosts, antiviral resistance, and immune evasion. The virus’s diversity challenges vaccine development, potentially generating numerous genetically distinct strains through reassortment.

During the past decades, it has been evident that IBV represents a considerable role in flu epidemics annually. The specific infection abilities of IBV in adolescents, the co-circulation of the two different lineages that cause persistent conflict between circulating strains and vaccine, along with the evolution of NAI-resistance-linked mutations and no possible fitness cost this further suggests the significance of IBV regarding human pathogens.

The existing anti-influenza drugs appear insufficient to provide a maximum therapeutic impact. Neuraminidase and viral polymerase inhibitors are two classes of antiviral drugs developed, examined, and permitted to be used. Some strains of IBVs show resistance to these inhibitors, as mentioned above. Among them, Baloxavir, a novel medicine in the market, shows effectiveness towards many oseltamivir-resistant strains and does not vary substantially in terms of the efficacy and safety of known drugs for the treatment of influenza. However, more research is needed, and it is highly advised that it be used it in a broader range of society’s socio-demographic factors and economic loss indications. Currently, the aim is to use rational antiviral treatment to lower the chance of choosing resistant strains, and to work with national reference laboratories to monitor influenza virus resistance.

## Data Availability

Data and materials will be provided upon request. If someone wants to request the data from this study, then the submitting author is responsible for providing the requested data and materials.

## Supplementary Materials

The following supporting information can be downloaded at: https://www.mdpi.com/article/10.3390/cimb46010014/s1, Figure S1: Economic impact of pandemic influenza in China; Table S1: The economic impact of pandemic influenza in Chicago, IL, USA.

## Abbreviations

IBV	Influenza B virus
WHO	World Health Organization
NA	Neuraminidase
HA	Hemagglutinin
PA	Acidic Polymerase
vRNP	Viral ribonucleoprotein
vRNA	Viral RNA
RBS	Receptor-binding site
LAIV	live attenuated influenza vaccine
NLS	Nuclear localization signal
TGN	Trans-Golgi network
RdRp	RNA-dependent RNA polymerase
CTD	C-terminal repeat domain
CRM	Chromosome Region Maintenance
GTP	Guanosine triphosphate
NAI	Neuraminidase Inhibitors
PIs	Viral polymerase inhibitors
bnAbs	Broadly neutralizing antibodies
RBS	Receptor binding site
HI	Hemagglutination inhibition
DADs	Direct-acting drugs
NTZ	Nitazoxanide
TIZ	Tizoxanide

## References

[1] Peasah S.K., Azziz-Baumgartner E., Breese J., Meltzer M.I., Widdowson M.-A. (2013). Influenza cost and cost-effectiveness studies globally—A review. Vaccine.

[2] Fischer W.A., Gong M., Bhagwanjee S., Sevransky J. (2014). Global Burden of Influenza as a Cause of Cardiopulmonary Morbidity and Mortality. Glob. Heart.

[3] Javanian M., Barary M., Ghebrehewet S., Koppolu V., Vasigala V.R., Ebrahimpour S. (2021). A brief review of influenza virus infection. J. Med. Virol..

[4] Niederman M.S., Krilov L.R. (2013). Acute lower respiratory infection in developing countries. Lancet.

[5] Nguyen Y.T., Graitcer S.B., Nguyen T.H., Tran D.N., Pham T.D., Le M.T., Tran H.N., Bui C.T., Dang D.T., Nguyen L.T. (2013). National surveillance for influenza and influenza-like illness in Vietnam, 2006–2010. Vaccine.

[6] Eisenstein M. (2019). Towards a universal flu vaccine. Nature.

[7] Grohskopf L.A. (2019). Prevention and control of seasonal influenza with vaccines: Recommendations of the Advisory Committee on Immunization Practices—United States, 2019–2020 influenza season. MMWR. Recomm. Rep..

[8] Nypaver C., Dehlinger C., Carter C. (2021). Influenza and influenza vaccine: A review. J. Midwifery Women’s Health.

[9] Francis M.E., King M.L., Kelvin A.A. (2019). Back to the future for influenza preimmunity—Looking back at influenza virus history to infer the outcome of future infections. Viruses.

[10] Tumpey T.M., Basler C.F., Aguilar P.V., Zeng H., Solórzano A., Swayne D.E., Cox N.J., Katz J.M., Taubenberger J.K., Palese P. (2005). Characterization of the reconstructed 1918 Spanish influenza pandemic virus. Science.

[11] Zimmer S.M., Burke D.S. (2009). Historical perspective—Emergence of influenza A (H1N1) viruses. N. Engl. J. Med..

[12] Nelson M.I., Viboud C., Simonsen L., Bennett R.T., Griesemer S.B., St George K., Taylor J., Spiro D.J., Sengamalay N.A., Ghedin E. (2008). Multiple reassortment events in the evolutionary history of H1N1 influenza A virus since 1918. PLoS Pathog..

[13] Jester B.J., Uyeki T.M., Jernigan D.B. (2020). Fifty years of influenza A (H3N2) following the pandemic of 1968. Am. J. Public Health.

[14] Broadbent A.J., Subbarao K. (2011). Influenza virus vaccines: Lessons from the 2009 H1N1 pandemic. Curr. Opin. Virol..

[15] Hannoun C. (2013). The evolving history of influenza viruses and influenza vaccines. Expert Rev. Vaccines.

[16] Koutsakos M., Nguyen T.H., Barclay W.S., Kedzierska K. (2016). Knowns and unknowns of influenza B viruses. Future Microbiol..

[17] Wang Q., Tian X., Chen X., Ma J. (2007). Structural basis for receptor specificity of influenza B virus hemagglutinin. Proc. Natl. Acad. Sci. USA.

[18] Gong H., Shen X., Yan H., Lu W.Y., Zhong G.J., Dong K.G., Yang J., Yu H.J. (2021). Estimating the disease burden of seasonal influenza in China, 2006–2019. Zhonghua Yi Xue Za Zhi.

[19] Dorratoltaj N., Marathe A., Lewis B.L., Swarup S., Eubank S.G., Abbas K.M. (2017). Epidemiological and economic impact of pandemic influenza in Chicago: Priorities for vaccine interventions. PLoS Comput. Biol..

[20] Ni F., Mbawuike I.N., Kondrashkina E., Wang Q. (2014). The roles of hemagglutinin Phe-95 in receptor binding and pathogenicity of influenza B virus. Virology.

[21] Burmeister W., Ruigrok R., Cusack S. (1992). The 2.2 A resolution crystal structure of influenza B neuraminidase and its complex with sialic acid. EMBO J..

[22] Burnham A.J., Baranovich T., Govorkova E.A. (2013). Neuraminidase inhibitors for influenza B virus infection: Efficacy and resistance. Antivir. Res..

[23] Demers A., Ran Z., Deng Q., Wang D., Edman B., Lu W., Li F. (2014). Palmitoylation is required for intracellular trafficking of influenza B virus NB protein and efficient influenza B virus growth in vitro. J. Gen. Virol..

[24] Fischer W.B., Pitkeathly M., Wallace B.A., Forrest L.R., Smith G.R., Sansom M.S.P. (2000). Transmembrane peptide NB of influenza B: A simulation, structure, and conductance study. Biochemistry.

[25] Tosteson M.T., Auld D.S., Tosteson D.C. (1989). Voltage-gated channels formed in lipid bilayers by a positively charged segment of the Na-channel polypeptide. Proc. Natl. Acad. Sci. USA.

[26] Hatta M., Kawaoka Y. (2003). The NB protein of influenza B virus is not necessary for virus replication in vitro. J. Virol..

[27] McCullers J.A., Hoffmann E., Huber V.C., Nickerson A.D. (2005). A single amino acid change in the C-terminal domain of the matrix protein M1 of influenza B virus confers mouse adaptation and virulence. Virology.

[28] Cao S., Jiang J., Li J., Li Y., Yang L., Wang S., Yan J., Gao G.F., Liu W. (2014). Characterization of the nucleocytoplasmic shuttle of the matrix protein of influenza B virus. J. Virol..

[29] Wang J., Pielak R.M., McClintock M.A., Chou J.J. (2009). Solution structure and functional analysis of the influenza B proton channel. Nat. Struct. Mol. Biol..

[30] Mould J.A., Paterson R.G., Takeda M., Ohigashi Y., Venkataraman P., Lamb R.A., Pinto L.H. (2003). Influenza B virus BM2 protein has ion channel activity that conducts protons across membranes. Dev. Cell.

[31] Zhang H., Yu H., Wang J., Zhang M., Wang X., Ahmad W., Duan M., Guan Z. (2015). The BM2 protein of influenza B virus interacts with p53 and inhibits its transcriptional and apoptotic activities. Mol. Cell. Biochem..

[32] Horvath C.M., Williams M.A., Lamb R.A. (1990). Eukaryotic coupled translation of tandem cistrons: Identification of the influenza B virus BM2 polypeptide. EMBO J..

[33] Hatta M., Kohlmeier C.K., Hatta Y., Ozawa M., Kawaoka Y. (2009). Region required for protein expression from the stop-start pentanucleotide in the M gene of influenza B virus. J. Virol..

[34] Powell M.L., Napthine S., Jackson R.J., Brierley I., Brown T.D.K. (2008). Characterization of the termination–reinitiation strategy employed in the expression of influenza B virus BM2 protein. RNA.

[35] Ng A.K.-L., Lam M.K.-H., Zhang H., Liu J., Au S.W.-N., Chan P.K.-S., Wang J., Shaw P.-C. (2012). Structural basis for RNA binding and homo-oligomer formation by influenza B virus nucleoprotein. J. Virol..

[36] Shen Y.-F., Chen Y.-H., Chu S.-Y., Lin M.-I., Hsu H.-T., Wu P.-Y., Wu C.-J., Liu H.-W., Lin F.-Y., Lin G. (2011). E339-R416 salt bridge of nucleoprotein as a feasible target for influenza virus inhibitors. Proc. Natl. Acad. Sci. USA.

[37] Wanitchang A., Narkpuk J., Jongkaewwattana A. (2013). Nuclear import of influenza B virus nucleoprotein: Involvement of an N-terminal nuclear localization signal and a cleavage-protection motif. Virology.

[38] Sherry L., Smith M., Davidson S., Jackson D. (2014). The N terminus of the influenza B virus nucleoprotein is essential for virus viability, nuclear localization, and optimal transcription and replication of the viral genome. J. Virol..

[39] Reich S., Guilligay D., Pflug A., Malet H., Berger I., Crépin T., Hart D., Lunardi T., Nanao M., Ruigrok R.W.H. (2014). Structural insight into cap-snatching and RNA synthesis by influenza polymerase. Nature.

[40] Wakai C., Iwama M., Mizumoto K., Nagata K. (2011). Recognition of cap structure by influenza B virus RNA polymerase is less dependent on the methyl residue than recognition by influenza A virus polymerase. J. Virol..

[41] Dauber B., Heins G., Wolff T. (2004). The influenza B virus nonstructural NS1 protein is essential for efficient viral growth and antagonizes beta interferon induction. J. Virol..

[42] Guan R., Ma L.-C., Leonard P.G., Amer B.R., Sridharan H., Zhao C., Krug R.M., Montelione G.T. (2011). Structural basis for the sequence-specific recognition of human ISG15 by the NS1 protein of influenza B virus. Proc. Natl. Acad. Sci. USA.

[43] Noah D.L., Twu K.Y., Krug R.M. (2003). Cellular antiviral responses against influenza A virus are countered at the posttranscriptional level by the viral NS1A protein via its binding to a cellular protein required for the 3′ end processing of cellular pre-mRNAS. Virology.

[44] Donelan N.R., Dauber B., Wang X., Basler C.F., Wolff T., García-Sastre A. (2004). The N-and C-terminal domains of the NS1 protein of influenza B virus can independently inhibit IRF-3 and beta interferon promoter activation. J. Virol..

[45] Iwasaki A., Pillai P.S. (2014). Innate immunity to influenza virus infection. Nat. Rev. Immunol..

[46] Paragas J., Talon J., O’Neill R.E., Anderson D.K., García-Sastre A., Palese P. (2001). Influenza B and C virus NEP (NS2) proteins possess nuclear export activities. J. Virol..

[47] Boulo S., Akarsu H., Ruigrok R.W., Baudin F. (2007). Nuclear traffic of influenza virus proteins and ribonucleoprotein complexes. Virus Res..

[48] Crow M., Deng T., Addley M., Brownlee G.G. (2004). Mutational analysis of the influenza virus cRNA promoter and identification of nucleotides critical for replication. J. Virol..

[49] Plotch S.J., Tomasz J., Krug R.M. (1978). Absence of detectable capping and methylating enzymes in influenza virions. J. Virol..

[50] Dhar R., Chanock R.M., Lai C.J. (1980). Nonviral oligonucleotides at the 5′ terminus of cytoplasmic influenza viral mRNA deduced from cloned complete genomic sequences. Cell.

[51] Li M.L., Rao P., Krug R.M. (2001). The active sites of the influenza cap-dependent endonuclease are on different polymerase subunits. EMBO J..

[52] Engelhardt O.G., Smith M., Fodor E. (2005). Association of the influenza A virus RNA-dependent RNA polymerase with cellular RNA polymerase II. J. Virol..

[53] Nayak D.P., Balogun R.A., Yamada H., Zhou Z.H., Barman S. (2009). Influenza virus morphogenesis and budding. Virus Res..

[54] Samji T. (2009). Influenza A: Understanding the viral life cycle. Yale J. Biol. Med..

[55] Imai M., Watanabe S., Odagiri T. (2003). Influenza B virus NS2, a nuclear export protein, directly associates with the viral ribonucleoprotein complex. Arch. Virol..

[56] Hoffmann E., Mahmood K., Yang C.-F., Webster R.G., Greenberg H.B., Kemble G. (2002). Rescue of influenza B virus from eight plasmids. Proc. Natl. Acad. Sci. USA.

[57] Smyk J.M., Szydłowska N., Szulc W., Majewska A. (2022). Evolution of Influenza Viruses—Drug Resistance, Treatment Options, and Prospects. Int. J. Mol. Sci..

[58] Świerczyńska M., Mirowska-Guzel D.M., Pindelska E. (2022). Antiviral drugs in influenza. Int. J. Environ. Res. Public Health.

[59] Tappenden P., Jackson R., Cooper K., Rees A., Simpson E., Read R., Nicholson K.G. (2009). Amantadine, oseltamivir, and zanamivir for the prophylaxis of influenza (including a review of existing guidance no. 67): A systematic review and economic evaluation. Health Technol. Assess..

[60] Sheu T.G., Deyde V.M., Okomo-Adhiambo M., Garten R.J., Xu X., Bright R.A., Butler E.N., Wallis T.R., Klimov A.I., Gubareva L.V. (2008). Surveillance for neuraminidase inhibitor resistance among human influenza A and B viruses circulating worldwide from 2004 to 2008. Antimicrob. Agents Chemother..

[61] Hurt A.C., Besselaar T.G., Daniels R.S., Ermetal B., Fry A., Gubareva L., Huang W., Lackenby A., Lee R.T., Lo J. (2016). Global update on the susceptibility of human influenza viruses to neuraminidase inhibitors, 2014–2015. Antivir. Res..

[62] Brown S.K., Tseng Y.-Y., Aziz A., Baz M., Barr I.G. (2022). Characterization of influenza B viruses with reduced susceptibility to influenza neuraminidase inhibitors. Antivir. Res..

[63] Higgins R.R., Beniprashad M., Chong-King E., Li Y., Bastien N., Low D.E., Gubbay J.B. (2012). Recovery of influenza B virus with the H273Y point mutation in the neuraminidase active site from a human patient. J. Clin. Microbiol..

[64] Kohno S., Kida H., Mizuguchi M., Hirotsu N., Ishida T., Kadota J., Shimada J., S-021812 Clinical Study Group (2011). Intravenous peramivir for treatment of influenza A and B virus infection in high-risk patients. Antimicrob. Agents Chemother..

[65] Hurt A.C. (2014). The epidemiology and spread of drug resistant human influenza viruses. Curr. Opin. Virol..

[66] Shetty A.K., Peek L.A. (2012). Peramivir for the treatment of influenza. Expert Rev. Anti-Infect. Ther..

[67] Wester A., Shetty A.K. (2016). Peramivir injection in the treatment of acute influenza: A review of the literature. Infect. Drug Resist..

[68] Shie J.J., Fang J.M. (2019). Development of effective anti-influenza drugs: Congeners and conjugates—A review. J. Biomed. Sci..

[69] Watanabe A., Chang S.C., Kim M.J., Chu D.W., Ohashi Y., MARVEL Study Group (2010). Long-acting neuraminidase inhibitor laninamivir octanoate versus oseltamivir for treatment of influenza: A double-blind, randomized, noninferiority clinical trial. Clin. Infect. Dis..

[70] Han N., Oh J.M., Kim I.W. (2020). Assessment of adverse events related to anti-influenza neuraminidase inhibitors using the FDA adverse event reporting system and online patient reviews. Sci. Rep..

[71] Takashita E. (2021). Influenza polymerase inhibitors: Mechanisms of action and resistance. Cold Spring Harb. Perspect. Med..

[72] Mifsud E.J., Hayden F.G., Hurt A.C. (2019). Antivirals targeting the polymerase complex of influenza viruses. Antivir. Res..

[73] Abed Y., Fage C., Checkmahomed L., Venable M.-C., Boivin G. (2020). Characterization of contemporary influenza B recombinant viruses harboring mutations of reduced susceptibility to baloxavir marboxil, in vitro and in mice. Antivir. Res..

[74] Lampejo T. (2020). Influenza and antiviral resistance: An overview. Eur. J. Clin. Microbiol. Infect. Dis..

[75] Shen C., Zhang M., Chen Y., Zhang L., Wang G., Chen J., Chen S., Li Z., Wei F., Chen J. (2019). An IgM antibody targeting the receptor binding site of influenza B blocks viral infection with great breadth and potency. Theranostics.

[76] Shen C., Chen J., Li R., Zhang M., Wang G., Stegalkina S., Zhang L., Chen J., Cao J., Bi X. (2017). A multimechanistic antibody targeting the receptor binding site potently cross-protects against influenza B viruses. Sci. Transl. Med..

[77] Broekhuysen J., Stockis A., Lins R., De Graeve J., Rossignol J. (2000). Nitazoxanide: Pharmacokinetics and metabolism in man. Int. J. Clin. Pharmacol. Ther..

[78] White A.C. (2004). Nitazoxanide: A new broad spectrum antiparasitic agent. Expert Rev. Anti-Infect. Ther..

[79] Rossignol J.F. (2014). Nitazoxanide: A first-in-class broad-spectrum antiviral agent. Antivir. Res..

[80] Koszalka P., Tilmanis D., Hurt A.C. (2017). Influenza antivirals currently in late-phase clinical trial. Influenza Other Respir. Viruses.

[81] Yang F., Pang B., Lai K.K., Cheung N.N., Dai J., Zhang W., Zhang J., Chan K.-H., Chen H., Sze K.-H. (2021). Discovery of a novel specific inhibitor targeting influenza A virus nucleoprotein with pleiotropic inhibitory effects on various steps of the viral life cycle. J. Virol..

[82] Uehara T., Shishido T., Ishibashi T. (2016). S-033188, a small molecule inhibitor of Cap-dependent endonuclease of influenza A and B virus, leads to rapid and profound viral load reduction. Options IX Control. Influenza.

[83] Skalickova S., Heger Z., Krejcova L., Pekarik V., Bastl K., Janda J., Kostolansky F., Vareckova E., Zitka O., Adam V. (2015). Perspective of use of antiviral peptides against influenza virus. Viruses.

[84] Sharma P.K., Bansal S., Banik A. (2011). Noninvasive routes of proteins and peptides drug delivery. Indian J. Pharm. Sci..

[85] Vlieghe P., Lisowski V., Martinez J., Khrestchatisky M. (2010). Synthetic therapeutic peptides: Science and market. Drug Discov. Today.

