# Supplementary material for: Natural History of Influenza B Virus—Current Knowledge on Treatment, Resistance and Therapeutic Options
Source: Curr Issues Mol Biol. 2023 Dec 26;46(1):183–99. doi: 10.3390/cimb46010014 (PMC10814056; doi:10.3390/cimb46010014)
Supplement: Supplementary file 1 [file cimb-46-00014-s001.zip › cimb-2705513-supplementary.pdf]

## Figure S1: Economic impact of pandemic influenza in China

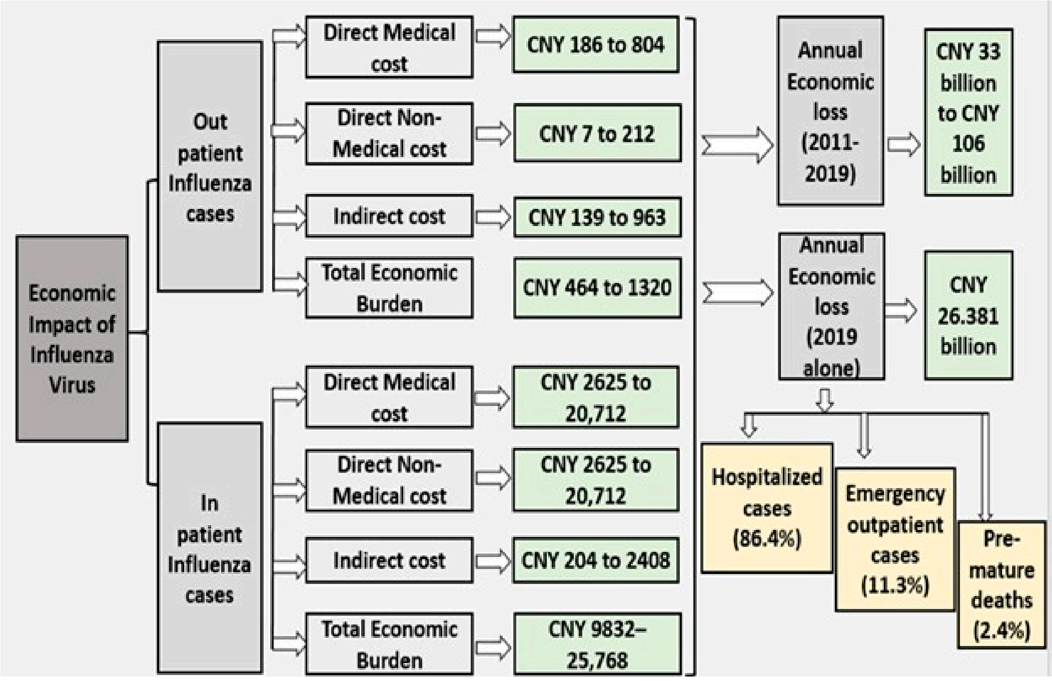


The total economic burden was 26.3g billion CNY, accounting for GDP in 2019: of which the hospitalization-related economic burden accounted for the highest proportion (86.40%, 22.79 billion •CNY): followed by the outpatient-related economic burden (11.3%: 2.97 billion CNY and the indirect economic burden of productivity Logs of premature deaths was the lowest (2.4 0.62 billion CNY) Largest economic burden observed m East China (10.51 billion CNY) and smallest observed in Northeast China (0.38 billion CNY) [19].

## Table S1: Economic impact of pandemic influenza in Chicago, USA

| Catastrophic influenza | Base case | Static model | Dynamic model |
|---|---|---|---|
| Pandemic cost per capita | $678.10 | $581.09 | $370.56 |
| Attack rate | 30.15%<br>[0–19 years: 48.35%]<br>[20–64 years: 23.94%]<br>[65+ years: 14.91%] | 25.33%<br>[0–19 years: 40.62%]<br>[20–64 years: 20.11%]<br>[65+ years: 12.52%] | 16.34%<br>[0–19 years: 27.97%]<br>[20–64 years: 12.25%]<br>[65+ years: 7.34%] |
| Reproduction number | 1.19 | 1.15 | 1.09 |
| **Strong influenza** | **Base case** | **Static model** | **Dynamic model** |
| Pandemic cost per capita | $486.67 | $420.28 | $90.81 |
| Attack rate | 21.96%<br>[0–19 years: 36.76%]<br>[20–64 years: 16.82%]<br>[65+ years: 10.18%] | 18.45%<br>[0–19 years: 30.88%]<br>[20–64 years: 14.13%]<br>[65+ years: 8.55%] | 3.90%<br>[0–19 years: 6.47%]<br>[20–64 years: 2.59%]<br>[65+ years: 1.52%] |
| Reproduction number | 1.13 | 1.11 | 1.02 |
| **Moderate influenza** | **Base case** | **Static model** | **Dynamic model** |
| Pandemic cost per capita | $255.18 | $225.83 | $14.85 |
| Attack rate | 11.73%<br>[0–19 years: 20.64%]<br>[20–64 years:8.55%]<br>[65+ years: 5.05%] | 9.85%<br>[0–19 years:17.34%]<br>[20–64 years: 7.19%]<br>[65+ years: 4.24%] | 0.16%<br>[0–19 years: 0.30%]<br>[20–64 years: 0.11%]<br>[65+ years: 0.06%] |

Table S1: cost per capita, attack rate, and reproduction number with and without vaccine intervention Ares presented for catastrophic, strong, and moderate Influenza pandemic scenarios. The vaccine intervention is implemented at 40% compliance and 40% efficacy which decreases the pandemic cost per capita, attack rate, and reproduction number. Pandemic cost per capita. attack rate and reproduction number are relatively lower in the dynamic model (direct indirect effects) in comparison to the static model (direct effect only) [20].
